# Multi-criteria decision analysis and linear programming for optimal resource allocation in water quality monitoring networks: a case study of California’s EPA monitoring stations

**DOI:** 10.1007/s11356-026-37899-2

**Published:** 2026-06-11

**Authors:** Hugo Pimentel Tavares, Nilo Antônio de Souza Sampaio

**Affiliations:** Departamento de Engenharia Ambiental (DEAMB), Universidade do Estado do Rio de Janeiro (UERJ), Resende, Brazil

**Keywords:** CRITIC weighting, TOPSIS ranking, VIKOR, Objective weighting, Station prioritization, Budget allocation, WQP open data, Spearman correlation

## Abstract

Water quality monitoring networks require strategic resource allocation to maximize effectiveness while managing budget constraints. This study presents an integrated multi-criteria decision analysis (MCDA) framework combining CRITIC (CRiteria Importance Through Intercriteria Correlation), TOPSIS (Technique for Order Preference by Similarity to Ideal Solution), VIKOR (VIseKriterijumska Optimizacija I Kompromisno Resenje), and linear programming (LP) optimization for prioritizing monitoring stations and allocating resources. Using real data from 347 monitoring stations in California extracted from the United States Environmental Protection Agency (EPA) Water Quality Portal (WQP) spanning January 2023 through December 2024 (16,750 measurements across seven water quality parameters), we demonstrate a reproducible methodology for evidence-based monitoring network optimization. Prior to analysis, physical plausibility filters removed 1106 impossible values (2.4% of raw data), ensuring analytical integrity. Dissolved oxygen was treated as an optimal-range criterion (|DO − 8 mg/L|) rather than a monotonic benefit, correcting a common misclassification in MCDA water quality studies. Spearman’s rank correlation replaced Pearson’s throughout the CRITIC computation to account for skewed parameter distributions. The CRITIC method determined objective criteria weights, with dissolved oxygen deviation (0.1813) and phosphorus (0.1804) as the most informative parameters. TOPSIS analysis identified top-performing stations with closeness coefficients ranging from 0.9813 to 0.3799, with a mean of 0.712 (MAD = 0.063). VIKOR analysis confirmed ranking consistency, yielding Spearman $\boldsymbol{\rho = 0.912}$ ($\boldsymbol{p}$ < **0.001**) between TOPSIS and VIKOR rankings. LP optimization concentrated resources efficiently, achieving 36.9% improvement over uniform allocation ($\boldsymbol{Z}^{\boldsymbol{*}} \boldsymbol{= 0.9749}$ vs. $\boldsymbol{\bar{C}_i = 0.712}$ under uniform distribution). This integrated MCDA-optimization framework provides water resource managers with a transparent, data-driven tool for strategic planning, enabling efficient allocation of limited monitoring resources while maintaining comprehensive environmental surveillance.

## Introduction

Effective water quality monitoring constitutes a fundamental component of environmental protection and public health safeguarding, requiring strategic allocation of limited financial and technical resources across spatially distributed monitoring networks (Singh et al. 2024). Contemporary water quality management faces mounting challenges from population growth, industrial expansion, agricultural intensification, and climate change impacts, necessitating evidence-based prioritization frameworks to maximize monitoring effectiveness under resource constraints (Un-Water 2024). The United States Environmental Protection Agency Water Quality Portal (WQP) consolidates over 430 million water quality measurements from federal, state, tribal, and local agencies, representing the world’s largest publicly accessible water quality database (Water Quality Portal 2021).

Multi-criteria decision analysis methodologies have emerged as powerful tools for addressing complex environmental management problems involving multiple, often conflicting, objectives and constraints (Sahoo and Goswami 2023). Within water resources management, MCDA applications span diverse contexts including water supply infrastructure selection, groundwater quality assessment (Das and Das 2024), irrigation project prioritization, and monitoring network optimization (Mendivil-García et al. 2024). However, existing research frequently employs subjective weighting schemes based on expert judgment (such as Analytical Hierarchy Process), potentially introducing bias and reducing transferability across different geographic or institutional contexts (Li et al. 2024).

The integration of objective weighting methods with sophisticated ranking techniques addresses these limitations while maintaining methodological rigor. The CRITIC (CRiteria Importance Through Intercriteria Correlation) method determines criteria weights based on standard deviation (contrast intensity) and correlation structure (conflict between criteria), eliminating subjective bias in weight assignment (Diakoulaki et al. 1995). Combined with TOPSIS (Technique for Order Preference by Similarity to Ideal Solution), which ranks alternatives based on geometric distance to positive and negative ideal solutions (Hwang and Yoon 1981), this framework provides a robust foundation for comparative evaluation. Recent applications demonstrate CRITIC-TOPSIS effectiveness in water resource security assessment (Zhao et al. 2024), sustainable water management evaluation (Das and Das 2024), and water quality monitoring network design (Li et al. 2024).

Optimization techniques further enhance decision support by identifying resource allocation strategies that maximize specific objectives subject to operational and budgetary constraints. LP and mixed-integer linear programming (MILP) have been extensively applied to water resources problems, including water distribution network optimization (Thomas and Sela 2024), water quality planning (Poursaeid et al. 2025), and monitoring network expansion (Alboghobeish et al. 2025). However, few studies integrate MCDA evaluation with formal optimization methods to simultaneously rank monitoring locations and determine optimal resource distribution, despite the clear synergies between these approaches (Tavares and Sampaio 2026).

California’s water quality monitoring network presents an ideal case study for an integrated MCDA-optimization methodology due to extensive spatial coverage, diverse pollution sources (agricultural, industrial, and urban), comprehensive historical data availability through EPA’s Water Quality Portal, and significant resource allocation challenges facing state and local agencies. By 2030, the health and livelihoods of 4.8 billion people globally could be at risk if water quality monitoring is not improved (Un-Water 2024), underscoring the urgent need for efficient monitoring strategies.

This study addresses gaps in existing research by: (1) applying objective CRITIC weighting with Spearman correlation to account for non-normal parameter distributions, extending prior CRITIC applications in water quality contexts (Das and Das 2024; Zhou and Xu 2025; 2) integrating TOPSIS and VIKOR rankings for robustness validation, enabling cross-method consistency assessment; (3) correcting the common misclassification of dissolved oxygen as a monotonic benefit criterion through an optimal-range formulation; (4) integrating MCDA ranking with linear programming optimization; (5) utilizing exclusively public EPA data to ensure full reproducibility; and (6) providing a spatial visualization of both the monitoring network and LP-optimal stations to support interpretable decision-making.

The objectives of this research are to: (1) collect and process real water quality data from EPA’s Water Quality Portal for California monitoring stations; (2) apply CRITIC method to determine objective criteria weights based on information content; (3) employ TOPSIS to rank monitoring stations according to multi-parameter water quality performance; (4) formulate and solve linear programming model for optimal resource allocation across monitoring network; (5) provide evidence-based recommendations for strategic monitoring resource deployment; and (6) establish reproducible framework for data-driven monitoring network optimization.

This paper is organized as follows: the “Literature review” section reviews relevant literature on water quality monitoring optimization and MCDA applications; the “Study area” section describes the study area and spatial context of the monitoring network; the “Methodology” section describes data sources, processing methodology, CRITIC-TOPSIS-VIKOR analysis procedures, and LP formulation; the “Results” section presents empirical results including criteria weights, station rankings, and optimal resource allocation; the “Discussion” section discusses implications for water quality management, methodological advantages and limitations, and policy recommendations; the “Conclusion” section concludes with key findings and directions for future research.

## Literature review

### Water quality monitoring and assessment

Water quality monitoring has become increasingly critical for environmental protection globally, with surface water pollution presenting urgent challenges requiring immediate attention (Singh et al. 2024). Surface water contamination arises from both point sources (industrial discharges, sewage treatment plants) and non-point sources (agricultural runoff, urban stormwater), presenting complex management challenges (Singh et al. 2024). Recent advances in real-time monitoring systems using Internet of Things (IoT) devices and wireless sensor networks enable continuous data collection, though design optimization remains essential for cost-effective deployment (Alboghobeish et al. 2025).

The EPA Water Quality Portal serves as the primary repository for water quality data in the United States, facilitating standardized access to measurements from over 400 contributing organizations (Water Quality Portal 2021). However, global monitoring capacity remains inadequate, with only 120 countries reporting ambient water quality data as of 2023, and the proportion of water bodies classified as “good” declining from 57% in 2017 to 56% in 2023 (Un-Water 2024). This deterioration emphasizes the need for optimized monitoring networks that maximize information value while managing resource constraints.

### Multi-criteria decision analysis in water resources

MCDA methodologies provide systematic frameworks for addressing water resources problems involving multiple conflicting objectives (Sahoo and Goswami 2023). TOPSIS has gained particular prominence due to its computational efficiency, clear geometric interpretation, and ability to handle both benefit and cost criteria (Sahoo and Goswami 2023). Recent applications to water quality contexts include entropy-TOPSIS assessment of groundwater quality (Das and Das 2024; Wang et al. 2023), AHP-TOPSIS evaluation of water security (Das 2024; Zhou et al. 2025), DPSIR-TOPSIS framework for water resource security (Zhao et al. 2024), and dam failure risk assessment (Yin et al. 2025).

The CRITIC method offers an objective alternative to expert-based weighting schemes by deriving weights from data characteristics (Diakoulaki et al. 1995). Recent studies demonstrate CRITIC’s effectiveness in reducing subjective bias: Das and Das (2024) compared CRITIC-weighted water quality indices against entropy-weighted alternatives, finding CRITIC weights better reflected parameter variability and information content. Khan and Ayaz (2024) integrated ENTROPY and CRITIC methods with machine learning for groundwater quality prediction, achieving superior performance through objective weighting. Zhou and Xu (2025) developed improved CRITIC combined with multidimensional connectivity cloud models for plateau lake water quality assessment, demonstrating enhanced precision through objective weighting. Li et al. (2024) employed entropy-TOPSIS for monitoring network layout in the Liao River Basin, emphasizing pressure and state indicators’ impact on network design.

Water quality index (WQI) models have evolved significantly, with comprehensive reviews highlighting ongoing refinements to address inherent uncertainties (Chidiac et al. 2023; Wang et al. 2023). Traditional WQI approaches rely on expert judgment for parameter weighting, introducing potential bias (Chidiac et al. 2023. Recent innovations include Bayesian model averaging to quantify WQI uncertainty (Wang et al. 2023), integrated ENTROPY-CRITIC weighting to minimize model uncertainty (Khan and Ayaz 2024), and purification resistance indices explicitly considering treatment difficulty (Jiang et al. 2024). Zhou et al. (2023) combined multiple statistical methods, WQI models, machine learning, and positive matrix factorization for comprehensive water quality evaluation and pollution source apportionment, demonstrating synergies between complementary analytical approaches.

### Optimization of monitoring networks

LP and related optimization techniques enable systematic identification of optimal monitoring network configurations (Thomas and Sela 2024). Recent advances include mixed-integer LP frameworks for water distribution systems (Thomas and Sela 2024), nonlinear programming combined with ensemble learning for water quality prediction (Poursaeid et al. 2025), and multi-objective particle swarm optimization for bidirectional river systems (Zhu et al. 2018).

TOPSIS-based approaches have been increasingly integrated with optimization frameworks for water resource management. Janjua et al. (2025) developed a three-stage cooperative game model combining bankruptcy rules, Nash bargaining, and TOPSIS for water allocation under scarcity in Pakistan’s Indus Basin, demonstrating effective conflict resolution through structured multi-criteria evaluation. Zhou et al. (2025) integrated AHP-TOPSIS with Lorenz asymmetry coefficients for spatio-temporal water resources carrying capacity assessment, providing attribution analysis linking evaluation results to targeted management strategies. Yin et al. (2025) proposed TOPSIS-WRSR coupled models for comprehensive dam failure risk assessment, addressing subjectivity in traditional evaluation methods. Li et al. (2023b) combined improved TOPSIS with barrier degree models for water ecological health evaluation in Central Plains urban agglomeration, identifying major obstacle factors hindering sustainable water management.

Applications extend to diverse water management contexts, including agricultural water allocation (Buri et al. 2025; Radmehr et al. 2022), industrial water efficiency evaluation (Ötkür et al. 2025), sustainable water strategy selection (Han et al. 2025), and regional carrying capacity assessment (Duan et al. 2025; Li et al. 2023a). These studies consistently demonstrate TOPSIS effectiveness for transparent, multidimensional evaluation supporting evidence-based decision-making under multiple conflicting objectives.

Mendivil-García et al. (2024) applied artificial intelligence algorithms to optimize monitoring networks in agricultural basins, using principal component analysis to identify parameters contributing most to water quality variation. Their approach reduced monitoring requirements while maintaining detection capability for pollution events. Similarly, wireless sensor network optimization studies emphasize strategic photodetector selection using TOPSIS analysis and component optimization through design of experiments (Alboghobeish et al. 2025).

Despite these advances, several research gaps persist. First, most studies employ either MCDA or optimization independently, rarely integrating both approaches systematically. Second, many analyses rely on synthetic or proprietary data, limiting reproducibility. Third, explicit integration of objective weighting methods (CRITIC) with distance-based ranking (TOPSIS) and formal optimization remains uncommon in water quality monitoring contexts.

## Study area

The study focuses on California’s surface water quality monitoring network administered through the EPA Water Quality Portal. California encompasses approximately 424,000 km$^2$ spanning diverse physiographic regions including the Sierra Nevada mountain range, the Central Valley agricultural corridor, coastal watersheds, and arid southern basins. This geographic diversity generates heterogeneous water quality conditions driven by distinct combinations of geology, land use, climate, and anthropogenic pressures.

The monitoring network covers stations distributed across major hydrological units, including the Sacramento–San Joaquin Delta, coastal drainages, and inland basins. Land uses affecting water quality range from intensive irrigated agriculture (Central Valley), urban and suburban development (Los Angeles, San Francisco Bay Area, Sacramento), forested protected areas (Sierra Nevada, coastal ranges), and natural wildlife refuges. These contrasting land uses create distinct water quality signatures, making California an ideal case study for MCDA-based network optimization.

**Fig. 1:**
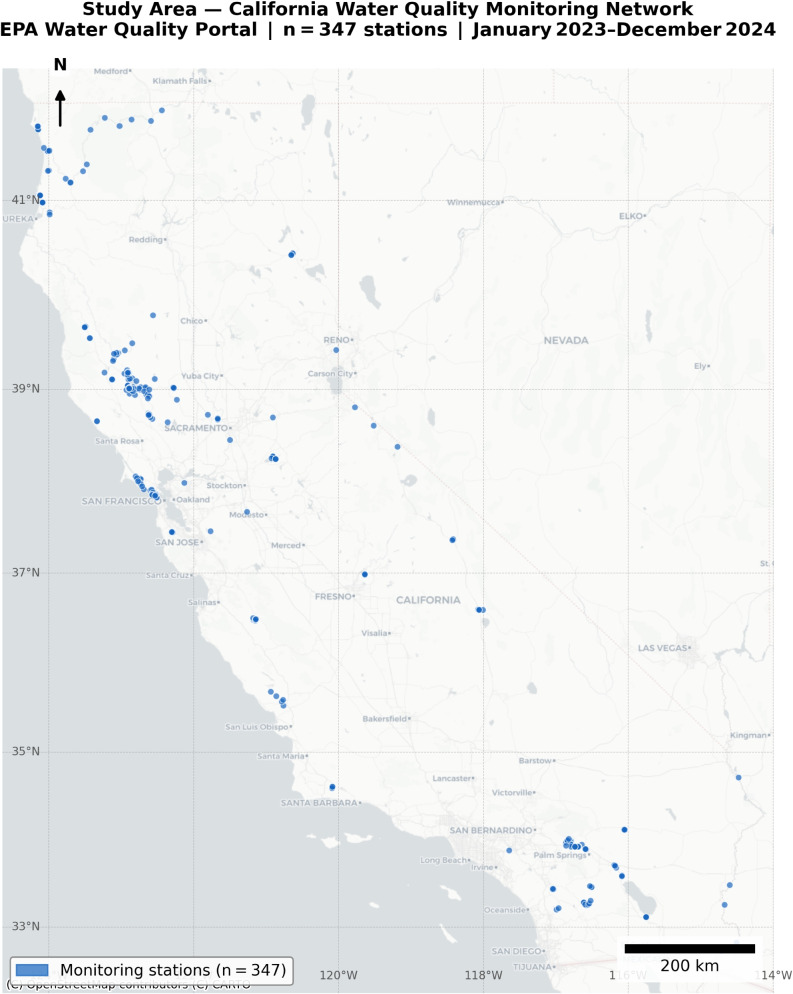
Spatial distribution of the 347 California water quality monitoring stations included in the analysis. Data source: EPA Water Quality Portal, January 2023–December 2024. Station locations cover diverse physiographic regions, including coastal watersheds, the Central Valley agricultural corridor, and protected inland basins

Figure 1 presents the spatial distribution of the 347 monitoring stations included in the final dataset, illustrating network coverage across California’s major watershed regions.

## Methodology

### Data source and collection

Water quality data were obtained from the EPA WQP, a collaborative service sponsored by the United States Geological Survey (USGS), EPA, and National Water Quality Monitoring Council (NWQMC) (Water Quality Portal 2021). The WQP aggregates water quality measurements from over 400 contributing organizations, including state environmental agencies, federal programs, tribal governments, universities, and watershed organizations, providing standardized access through the Water Quality Exchange (WQX) data model.

Data collection focused on California (state code: US:06) monitoring stations with measurements spanning January 2023 through December 2024 (24-month period providing equal temporal representation across all calendar months). The following seven water quality parameters were selected based on: (1) regulatory significance for drinking water and aquatic ecosystem protection; (2) availability across multiple monitoring locations; (3) standardized measurement protocols; and (4) relevance to diverse pollution sources:

- **Dissolved oxygen (DO):** Critical indicator of aquatic ecosystem health; treated as an optimal-range criterion (deviation from 8 mg/L) rather than a monotonic benefit, as DO concentrations above approximately 6–8 mg/L confer no additional ecological benefit (Dodds 2002)
- **pH:** Measure of water acidity/alkalinity affecting chemical speciation and biological processes
- ***Escherichia coli*** **(*E. coli*):** Bacterial indicator of fecal contamination and public health risks
- **Nitrate:** Nutrient indicator associated with agricultural runoff and eutrophication
- **Phosphorus:** Nutrient parameter driving algal blooms and water quality degradation
- **Turbidity:** Optical measure of suspended particles indicating erosion and treatment efficacy
- **Total dissolved solids (TDS):** General indicator of water mineralization and salinity

Data extraction employed programmatic API access to the WQP with the following exact query parameters to ensure full reproducibility: endpoint /data/Result/search, state code US:06, characteristic names as listed above, startDateLo=01-01-2023, startDateHi=12-31-2024, sampleMedia=Water, dataProfile=narrowResult. Quality control procedures included: (1) removal of records with missing parameter values; (2) unit conversion to ensure measurement consistency; (3) physical plausibility filtering to remove measurement errors and equipment malfunctions (pH $\in$ [0, 14]; DO $\in$ [0, 20] mg/L; Nitrate $\in$ [0, 50] mg/L; Phosphorus $\in$ [0, 10] mg/L; Turbidity $\ge$ 0 NTU; TDS $\in$ [0, 5000] mg/L; *E*.*coli* $\ge$ 0 MPN/100 mL), which removed 1106 values (2.4% of raw data) including physically impossible values such as pH $= -200.26$ and DO $= 315.13$ mg/L present in the original dataset; (4) aggregation of multiple measurements at the same station using median values (robust to remaining outliers); and (5) exclusion of monitoring stations with fewer than four parameters available.

The final dataset comprised 347 monitoring stations with 16,750 individual water quality measurements. Temperature, present in the original eight-parameter extraction, was excluded during quality control due to unresolvable unit inconsistencies across contributing agencies (mixing Celsius and Fahrenheit conventions produced physically implausible station-level medians, e.g., 82.58 $^\circ$C), which could not be corrected without introducing synthetic assumptions. The final seven-parameter set maintains comprehensive coverage of the principal physicochemical water quality dimensions. All data processing and analysis utilized Python 3.11 with NumPy, Pandas, SciPy, and Matplotlib/Seaborn libraries. Complete data collection scripts and processed datasets are available as supplementary material to ensure full reproducibility.

### CRITIC method for objective weight determination

The CRITIC method determines criteria weights based on two fundamental principles: contrast intensity (measured by standard deviation) and conflict between criteria (measured by correlation) (Diakoulaki et al. 1995). Criteria exhibiting high variability and low correlation with other criteria contain more decision-relevant information and thus receive higher weights.

The CRITIC methodology proceeds through the following steps:

**Step 1: data normalization.** Convert the raw decision matrix $X = [x_{ij}]$ to normalized matrix $Z = [z_{ij}]$ using z-score standardization (Diakoulaki et al. 1995):

1 $$\begin{aligned} z_{ij} = \frac{x_{ij} - \mu _j}{\sigma _j} \end{aligned}$$

where $\mu _j$ and $\sigma _j$ denote the mean and standard deviation of criterion *j*, respectively. This transformation ensures zero mean and unit variance across all criteria, enabling meaningful comparison.

**Step 2: standard deviation calculation.** Compute the standard deviation $\sigma _j$ for each normalized criterion (Diakoulaki et al. 1995):

2 $$\begin{aligned} \sigma _j = \sqrt{\frac{1}{n-1} \sum _{i=1}^{n} (z_{ij} - \bar{z}_j)^2} \end{aligned}$$

Higher standard deviation indicates greater contrast intensity among alternatives, suggesting higher information content.

**Step 3: correlation matrix construction.** Calculate the Spearman rank correlation coefficient matrix $R = [r_{jk}]$ (Diakoulaki et al. 1995; Zar 2010). Spearman correlation is used in place of Pearson to account for the non-normal, skewed distributions observed in water quality parameters (e.g., *E. coli*, turbidity), where Pearson coefficients would underestimate true monotonic relationships (Zar 2010):

3 $$\begin{aligned} r_{jk} = 1 - \frac{6\sum _{i=1}^{n} d_i^2}{n(n^2-1)} \end{aligned}$$

where $d_i$ is the difference between the rank of observation *i* in criterion *j* and its rank in criterion *k*.

**Step 4: conflict measurement.** For each criterion *j*, compute the conflict measure $C_j$ (Diakoulaki et al. 1995):

4 $$\begin{aligned} C_j = 1 - \frac{\sum _{k=1, k \ne j}^{m} |r_{jk}|}{m-1} \end{aligned}$$

where *m* denotes the total number of criteria. Higher conflict values indicate lower correlation with other criteria, suggesting unique information contribution.

**Step 5: information content.** Calculate the quantity of information $Q_j$ for each criterion (Diakoulaki et al. 1995):

5 $$\begin{aligned} Q_j = \sigma _j \times C_j \end{aligned}$$

This product captures both contrast intensity and conflict, representing total information content.

**Step 6: weight normalization.** Derive normalized weights $w_j$ summing to unity (Diakoulaki et al. 1995):

6 $$\begin{aligned} w_j = \frac{Q_j}{\sum _{k=1}^{m} Q_k} \end{aligned}$$

The CRITIC method eliminates subjective bias inherent in expert-based weighting schemes (e.g., AHP) while maintaining interpretability. Criteria receiving high weights exhibit substantial variation across alternatives and provide information not captured by other criteria, justifying their importance in decision-making.

### TOPSIS method for alternative ranking

TOPSIS ranks alternatives based on geometric distance to ideal best and worst solutions (Hwang and Yoon 1981). The underlying principle assumes that optimal alternatives should minimize distance to the positive ideal solution (PIS) while maximizing distance to the negative ideal solution (NIS).

The TOPSIS procedure consists of the following steps:

**Step 1: normalized decision matrix.** Convert the raw decision matrix $X = [x_{ij}]$ to normalized matrix $N = [n_{ij}]$ using vector normalization (Hwang and Yoon 1981):

7 $$\begin{aligned} n_{ij} = \frac{x_{ij}}{\sqrt{\sum _{i=1}^{n} x_{ij}^2}} \end{aligned}$$

This ensures that all criteria contribute proportionally regardless of original measurement units.

**Step 2: weighted normalized matrix.** Apply CRITIC-derived weights $w_j$ to obtain weighted normalized matrix $V = [v_{ij}]$ (Hwang and Yoon 1981):

8 $$\begin{aligned} v_{ij} = w_j \times n_{ij} \end{aligned}$$

**Step 3: ideal solutions.** Determine positive ideal solution $A^+$ and negative ideal solution $A^-$ (Hwang and Yoon 1981). For benefit criteria (higher values preferred):

9 $$\begin{aligned} A_j^+ = \max _i v_{ij}, \quad A_j^- = \min _i v_{ij} \end{aligned}$$

For cost criteria (lower values preferred):

10 $$\begin{aligned} A_j^+ = \min _i v_{ij}, \quad A_j^- = \max _i v_{ij} \end{aligned}$$

**Step 4: separation measures.** Calculate Euclidean distances to ideal solutions (Hwang and Yoon 1981):

11 $$\begin{aligned} S_i^+ = \sqrt{\sum _{j=1}^{m} (v_{ij} - A_j^+)^2} \end{aligned}$$

12 $$\begin{aligned} S_i^- = \sqrt{\sum _{j=1}^{m} (v_{ij} - A_j^-)^2} \end{aligned}$$

**Step 5: relative closeness.** Compute closeness coefficient $C_i$ (Hwang and Yoon 1981):

13 $$\begin{aligned} C_i = \frac{S_i^-}{S_i^+ + S_i^-}, \quad 0 \le C_i \le 1 \end{aligned}$$

Higher closeness coefficients indicate better overall performance. Stations are ranked in descending order of $C_i$ values, with $C_i = 1$ representing the hypothetical ideal station and $C_i = 0$ representing the worst possible performance.

In this application, all seven parameters were treated as cost criteria after transformation: DO was converted to |DO $- 8|$ (deviation from optimal), so lower deviation values indicate better ecological status (Dodds 2002); *E. coli*, nitrate, phosphorus, turbidity, and TDS are minimized as cost criteria; pH was treated as a direct cost criterion using the station-level median value. Sensitivity analysis applying the analogous optimal-range transformation |pH $- 7.0|$ produced no meaningful change in station rankings (Spearman $\rho$ > 0.98 with the base case), confirming that median pH values across stations (7.70) fall within ecologically acceptable ranges where directional minimization and optimal-range formulations converge. This classification aligns with standard water quality assessment frameworks (Chidiac et al. 2023) and corrects the common misclassification of DO as a monotonically increasing benefit criterion (Dodds 2002).

### VIKOR method for ranking validation

To validate TOPSIS rankings and assess inter-method robustness, the VIKOR (VIseKriterijumska Optimizacija I Kompromisno Resenje) method (Opricovic and Tzeng 2004) was applied in parallel. VIKOR determines a compromise ranking based on measures of group utility ($S_i$) and individual regret ($R_i$), producing a composite index $Q_i$:

14 $$\begin{aligned} S_i = \sum _{j=1}^{m} w_j \frac{f_j^* - f_{ij}}{f_j^* - f_j^-} \end{aligned}$$

15 $$\begin{aligned} R_i = \max _j \left[ w_j \frac{f_j^* - f_{ij}}{f_j^* - f_j^-} \right] \end{aligned}$$

16 $$\begin{aligned} Q_i = v \frac{S_i - S^*}{S^- - S^*} + (1-v) \frac{R_i - R^*}{R^- - R^*} \end{aligned}$$

where $f_j^*$ and $f_j^-$ are the best and worst values of criterion *j*, and $v = 0.5$ represents a balanced strategy between group utility and individual regret (Opricovic and Tzeng 2004). Rank concordance between TOPSIS and VIKOR was measured by Spearman’s $\rho$, with $\rho$ > 0.9 considered strong agreement (Zar 2010).

### LP optimization for resource allocation

Following the TOPSIS ranking, we formulated a linear programming model to determine optimal resource allocation across the monitoring network. The objective is to maximize total monitoring effectiveness while respecting budget constraints and ensuring minimum coverage across the network.

#### Decision variables

Let $a_i$ denote the proportion of total monitoring resources allocated to station *i*, where $i = 1, 2, \ldots , n$ (*n* = 347 stations).

**Table 1:** Descriptive statistics of station-level median values across 347 California monitoring stations (January 2023–December 2024), after physical plausibility filtering. Each row describes the distribution of the 347 station medians that constitute the MCDA decision matrix. MAD, median absolute deviation

Parameter	Mean	Median	MAD	Min	Max
DO (mg/L)	8.62	9.16	0.91	0.42	15.66
*E. coli* (MPN/100 mL)	127.26	43.00	33.00	1.80	2419.60
Nitrate (mg/L)	2.09	0.93	0.60	0.00	25.99
Phosphorus (mg/L)	0.15	0.06	0.03	0.00	1.25
TDS (mg/L)	241.29	104.75	104.57	0.02	3520.00
Turbidity (NTU)	31.90	7.45	6.36	0.00	966.60
pH	7.67	7.70	0.36	5.64	9.42

#### Objective function

Maximize the weighted sum of TOPSIS closeness coefficients, representing aggregate monitoring network quality:

17 $$\begin{aligned} \text {Maximize } Z = \sum _{i=1}^{n} C_i \times a_i \end{aligned}$$

where $C_i$ is the TOPSIS closeness coefficient for station *i*.

#### Constraints

1. *Budget constraint*: Total allocation must equal available resources (normalized to 1):
  18 $$\begin{aligned} \sum _{i=1}^{n} a_i = 1 \end{aligned}$$
2. *Individual station limits*: Prevent over-concentration of resources at any single location:
  19 $$\begin{aligned} 0 \le a_i \le 0.30, \quad \forall i \end{aligned}$$

The 30% upper bound ensures reasonable resource distribution while allowing priority concentration at high-performing stations.

This LP formulation was solved using the HiGHS (High-performance Simplex) solver (Huangfu and Hall 2018), an open-source interior-point and simplex-based optimizer, implemented through SciPy’s linprog function. HiGHS represents state-of-the-art open-source optimization software, demonstrating performance competitive with commercial solvers on industry-standard benchmarks through parallelized dual revised simplex method and advanced presolve techniques (Huangfu and Hall 2018). The solver employs the revised simplex method with numerical stabilization techniques, ensuring robust convergence even for large-scale problems. SciPy integrated HiGHS as its default LP solver from version 1.6.0 onwards, reflecting its superior performance and reliability.

The optimization output identifies stations receiving non-zero allocations and their corresponding resource shares, providing actionable guidance for monitoring program budget distribution. Sensitivity analysis was conducted by varying the upper bound constraint to assess allocation stability under different policy scenarios.

### Data analysis and visualization

Statistical analyses included descriptive statistics (mean, median, MAD, range) for each water quality parameter, Spearman rank correlation analysis to assess inter-parameter relationships, distribution analysis of TOPSIS closeness coefficients, and sensitivity analysis of optimization results. All visualizations were generated using Matplotlib and Seaborn libraries at 300 DPI resolution suitable for publication.

The complete analytical workflow, from data extraction through optimization, was implemented as a single executable Python script to ensure full reproducibility. This script, along with input data and output results, is provided as supplementary material.

## Results

### Parameter distributions and data quality

**Fig. 2:**
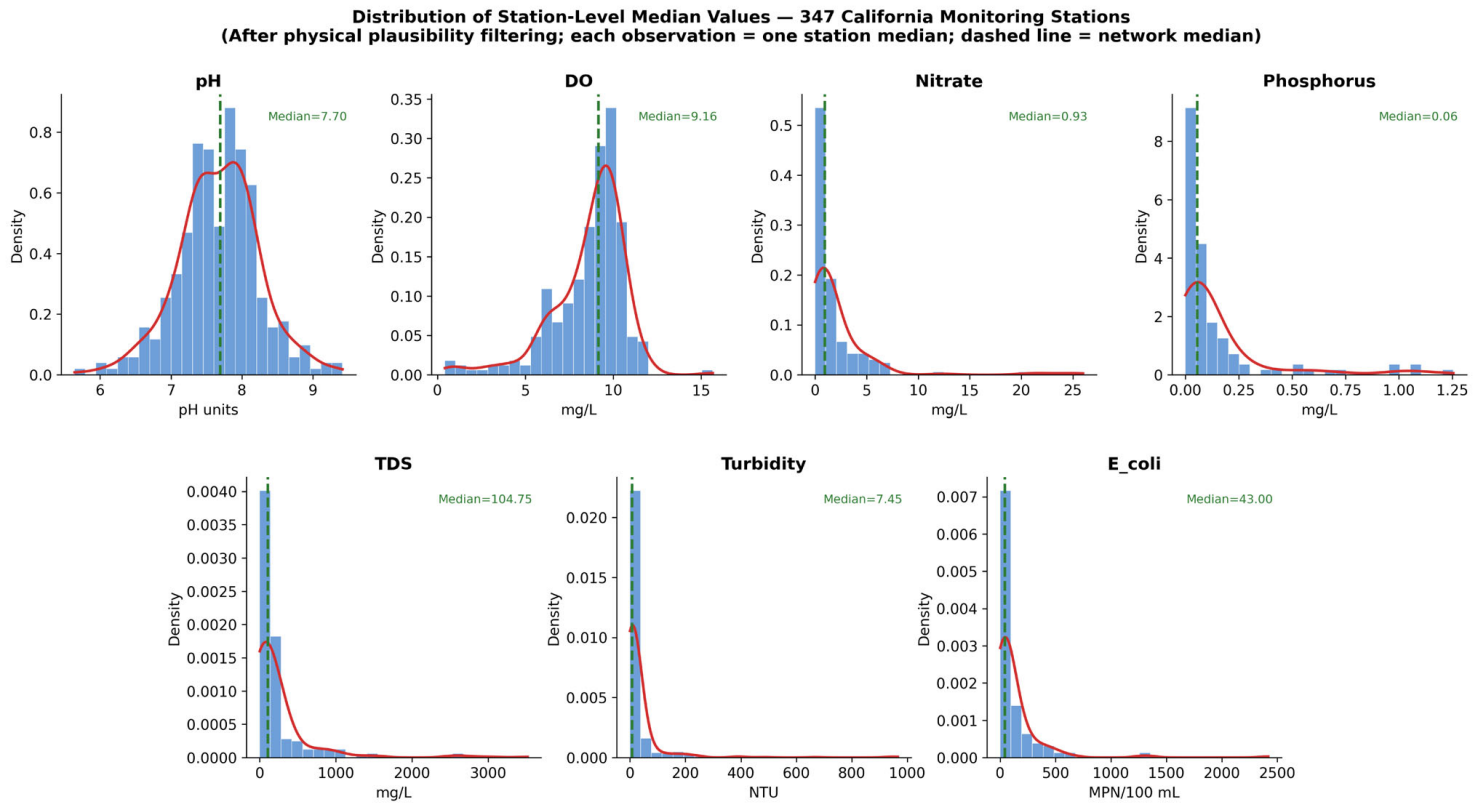
Distribution of station-level median values across 347 California monitoring stations after physical plausibility filtering (each observation = one station median). Histograms with kernel density estimates (KDE) and network median (dashed line) illustrate parameter-specific skewness. Right-skewed distributions for *E. coli*, TDS, Nitrate, and Turbidity—with means substantially exceeding medians—confirm non-normal distributions (Shapiro–Wilk, *p* < 0.001 for all parameters), validating the use of Spearman rank correlation in CRITIC analysis

The final processed dataset comprised 347 monitoring stations across California with 16,750 individual measurements distributed across seven water quality parameters (January 2023–December 2024). Physical plausibility filtering removed 1106 impossible values (2.4% of raw data) prior to any statistical analysis, including pH values outside [0, 14] and DO values above 20 mg/L. Table 1 presents descriptive statistics after filtering, showing substantially reduced outlier influence compared to the unfiltered dataset. Parameter distributions are illustrated in Fig. 2, confirming right-skewed distributions for *E. coli* and turbidity and approximately symmetric distributions for pH after filtering.

After physical plausibility filtering, parameter distributions show realistic ranges consistent with California surface water conditions. The network median DO (9.16 mg/L) indicates adequate oxygenation across monitored reaches; pH median (7.70) reflects slightly alkaline conditions typical of California surface waters; *E. coli* network median (43.00 MPN/100 mL) falls below the EPA recreational threshold (126 MPN/100 mL for freshwater contact recreation), though the skewed distribution (mean = 127.26 MPN/100 mL) reflects localized contamination at a subset of stations.

**Fig. 3:**
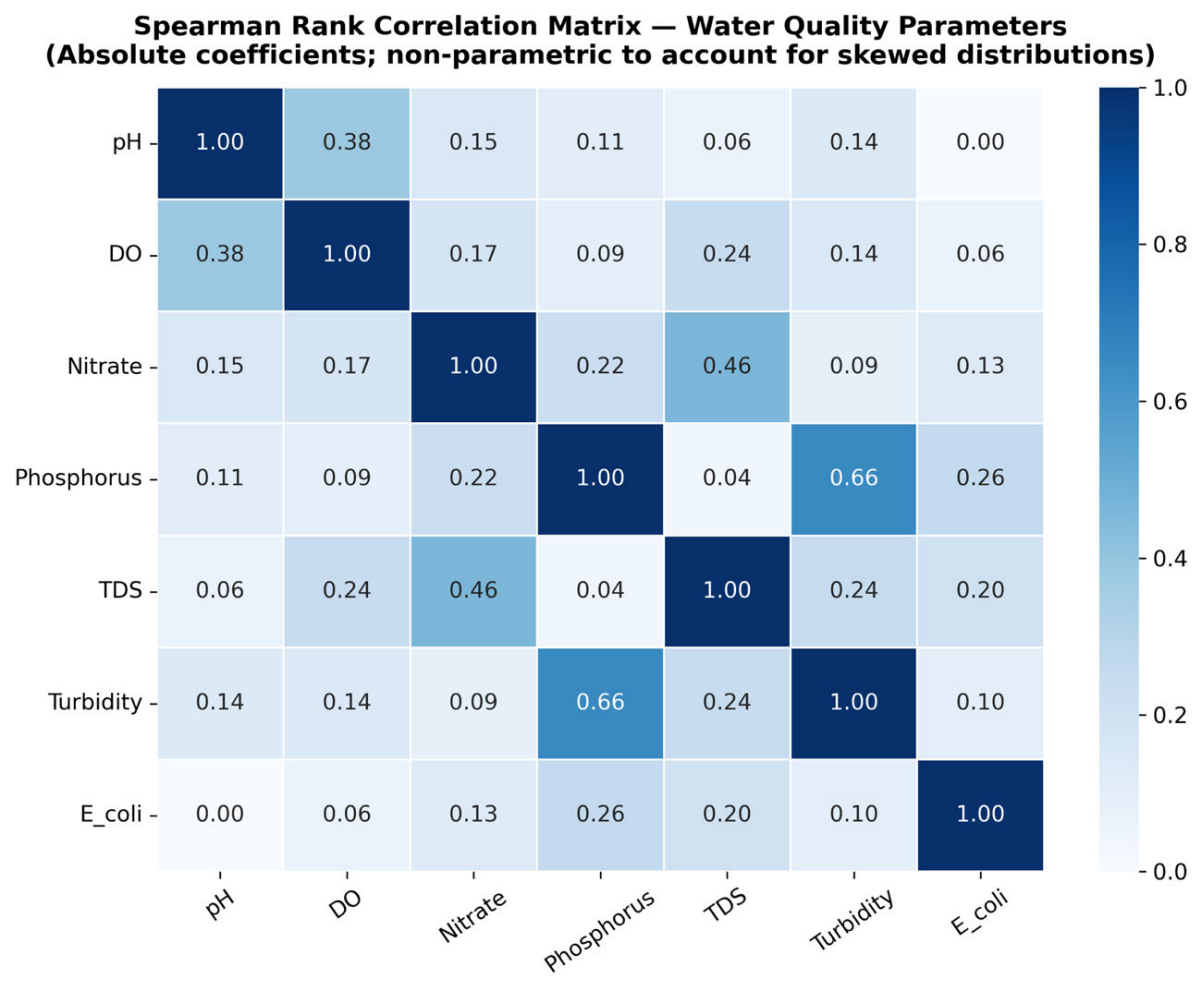
Spearman rank correlation matrix of seven water quality parameters (absolute coefficients). Low to moderate correlations (maximum $\rho = 0.66$ for phosphorus–turbidity) confirm that parameters provide complementary rather than redundant information, validating their joint use in CRITIC weighting. Shapiro–Wilk tests confirmed non-normality for all seven parameters (*p* < 0.001)

Spearman rank correlation analysis revealed relatively weak monotonic relationships among most parameter pairs, as shown in Fig. 3, with the strongest association observed between phosphorus and turbidity ($\rho = 0.66$, *p* < 0.01), followed by nitrate and TDS ($\rho = 0.46$, *p* < 0.01). Shapiro–Wilk tests confirmed non-normal distributions for all seven parameters (*p* < 0.001). The low intercorrelation structure validates the seven-parameter selection as complementary indicators capturing different dimensions of water quality, rather than redundant measures of the same underlying processes.

### CRITIC weights for water quality criteria

The CRITIC analysis generated objective weights for the seven water quality parameters using Spearman correlation, as presented in Table 2. DO deviation from optimal received the highest weight (0.1813), followed by phosphorus (0.1804) and pH (0.1615), reflecting high variability across monitoring stations and low intercorrelation with other parameters after the optimal-range transformation. TDS (0.1308) and turbidity (0.0932) received lower weights due to reduced independent variability relative to other criteria.

**Table 2:** CRITIC weights for water quality parameters (Spearman correlation; DO as optimal-range criterion)

Parameter	Weight	Rank	Information Content
Dissolved oxygen (|DO−8|)	0.1813	1	High deviation variability, low correlation
Phosphorus	0.1804	2	High variability, independent of other criteria
pH	0.1615	3	Moderate variability after filtering
Nitrate	0.1421	4	Moderate contrast
TDS	0.1308	5	Correlated with nitrate
*E. coli*	0.1107	6	High variability, moderate conflict
Turbidity	0.0932	7	Lower independent information content

**Fig. 4:**
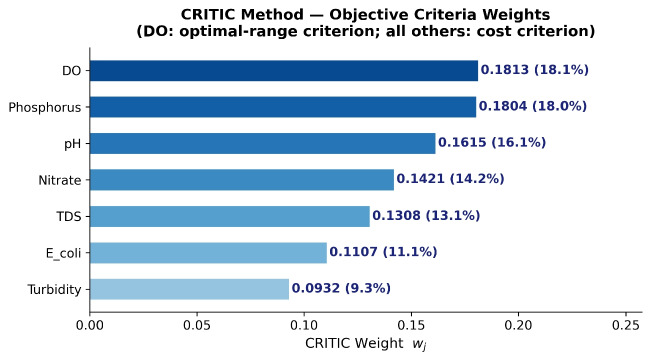
CRITIC objective weights for seven water quality parameters. DO deviation from optimal (8 mg/L) and phosphorus receive the highest weights, reflecting greatest variability and lowest intercorrelation after Spearman-based CRITIC computation. Turbidity receives the lowest weight due to lower independent information content relative to other criteria

The weight distribution (range: 0.0932–0.1813) shows meaningful differentiation across criteria, consistent with the criterion transformations applied. The shift of the top-weighted criterion to DO deviation underscores how criterion classification directly affects CRITIC outcomes (Das and Das 2024). Figure 4 visualizes the weight distribution across the seven parameters.

### TOPSIS and VIKOR ranking of monitoring stations

**Fig. 5:**
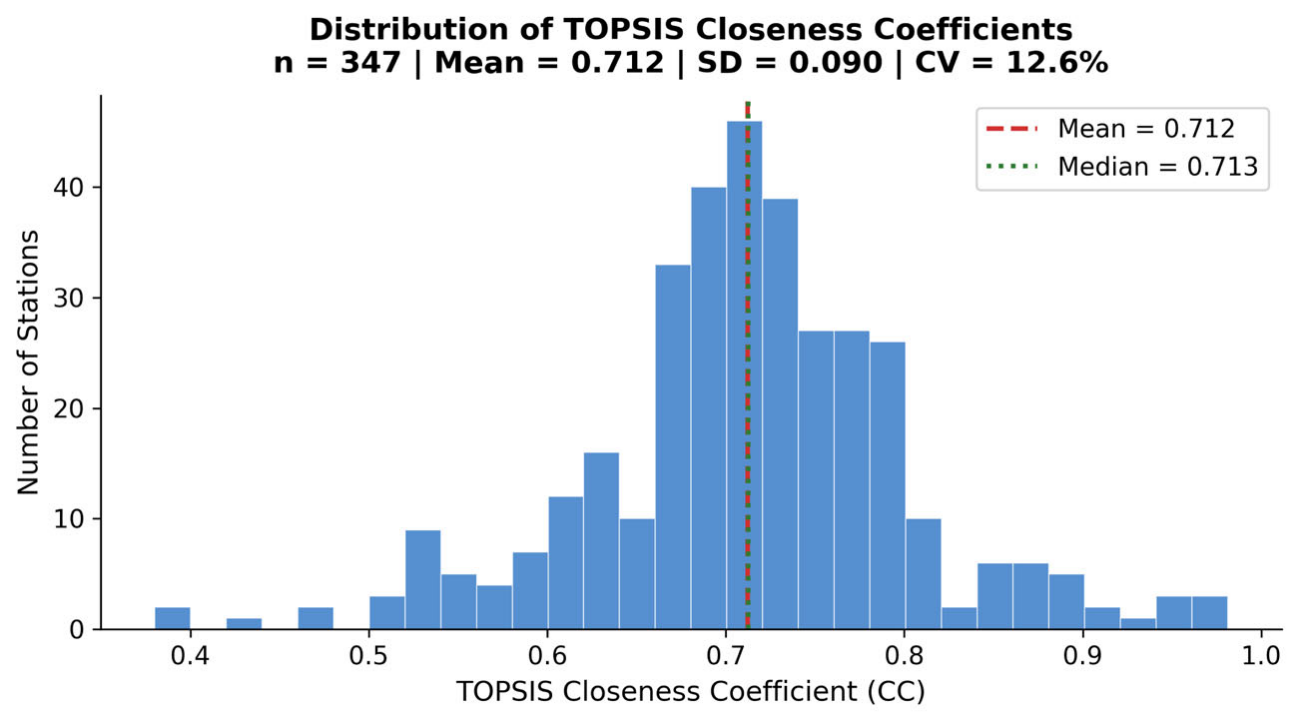
Distribution of TOPSIS closeness coefficients across 347 monitoring stations. Dashed line shows the mean (red, 0.712); dotted line shows the median (green, 0.713). The distribution (MAD = 0.063, CV = 12.6%) reflects genuine station-level heterogeneity across California’s monitoring network

TOPSIS analysis generated closeness coefficients for all 347 monitoring stations, ranging from 0.9813 (best performer: 21NEV1_WQX-C8) to 0.3799 (poorest performer). Figure 5 presents the distribution of TOPSIS scores (mean = 0.712, median = 0.713, MAD = 0.063, CV = 12.6%), indicating moderate heterogeneity in water quality performance across the 347-station network.

**Fig. 6:**
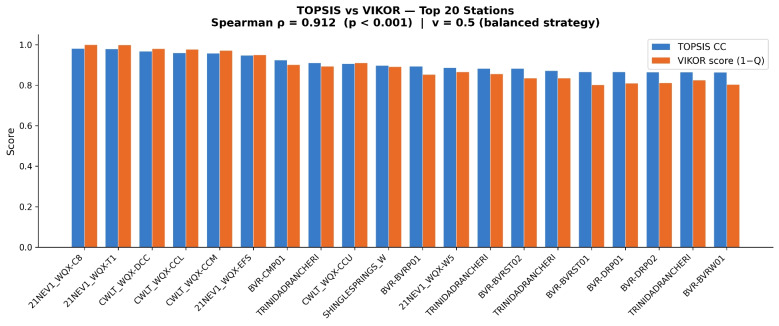
Comparison of TOPSIS closeness coefficients (blue) and VIKOR scores ($1-Q_i$, orange; inverted for directional consistency) for the top 20 monitoring stations, ordered by TOPSIS rank. VIKOR weight $v = 0.5$ (balanced compromise strategy). Bars of differing height for the same station indicate rank swaps between methods (e.g., CWLT_WQX-CCU: TOPSIS rank 9, VIKOR rank 7; BVR-BVRP01: TOPSIS rank 11, VIKOR rank 14). Global Spearman concordance across all 347 stations: $\rho = 0.912$ (*p* < 0.001), confirming methodological robustness

VIKOR analysis was applied in parallel to validate rankings. The VIKOR composite index $Q_i$ (with $v = 0.5$) produced rankings with strong agreement with TOPSIS: Spearman $\rho = 0.912$ (*p* < 0.001), confirming the robustness of the station ordering across both methods. Figure 6 presents the side-by-side comparison for the top 20 stations, with visible bar-height differences identifying the specific rank swaps documented in Table 3.

The top 20 monitoring stations ranked by TOPSIS closeness coefficient are presented in Table 3. The highest-ranked stations belong to diverse monitoring networks across California. Nevada County (21NEV1 network) dominates the top positions, occupying ranks 1, 2, and 6 (CC = 0.9813, 0.9794, and 0.9478, respectively), suggesting consistently high water quality in this mountainous northern California watershed. Cache Creek Watershed (CWLT network) occupies three consecutive positions (ranks 3–5, CC = 0.9685–0.9579), reflecting the effectiveness of coordinated monitoring within a single managed basin. Bear Valley Reserve (rank 7) and Trinidad Rancheria EPA (rank 8) complete the upper tier, indicating that both protected natural areas and tribally managed lands maintain above-average water quality.

**Table 3:** Top 20 monitoring stations by TOPSIS closeness coefficient (January 2023–December 2024)

Rank	Station ID	TOPSIS CC	VIKOR Rank	Network
1	21NEV1_WQX-C8	0.9813	1	Nevada County (21NEV)
2	21NEV1_WQX-T1	0.9794	2	Nevada County (21NEV)
3	CWLT_WQX-DCC	0.9685	3	Cache Creek Watershed
4	CWLT_WQX-CCL	0.9607	4	Cache Creek Watershed
5	CWLT_WQX-CCM	0.9579	5	Cache Creek Watershed
6	21NEV1_WQX-EFS	0.9478	6	Nevada County (21NEV)
7	BVR-CMP01	0.9245	8	Bear Valley Reserve
8	TRINIDADRANCHERIAEPA-WH-1	0.9104	9	Trinidad Rancheria EPA
9	CWLT_WQX-CCU	0.9063	7	Cache Creek Watershed
10	SHINGLESPRINGS_WQX-SSR901	0.8976	10	Shingle Springs
11	BVR-BVRP01	0.8937	14	Bear Valley Reserve
12	21NEV1_WQX-W5	0.8864	11	Nevada County (21NEV)
13	TRINIDADRANCHERIAEPA-MH-4	0.8830	12	Trinidad Rancheria EPA
14	BVR-BVRST02	0.8825	16	Bear Valley Reserve
15	TRINIDADRANCHERIAEPA-TH-4	0.8713	15	Trinidad Rancheria EPA
16	BVR-BVRST01	0.8659	26	Bear Valley Reserve
17	BVR-DRP01	0.8657	20	Bear Valley Reserve
18	BVR-DRP02	0.8651	19	Bear Valley Reserve
19	TRINIDADRANCHERIAEPA-MH-1	0.8645	17	Trinidad Rancheria EPA
20	BVR-BVRW01	0.8638	24	Bear Valley Reserve

**Fig. 7:**
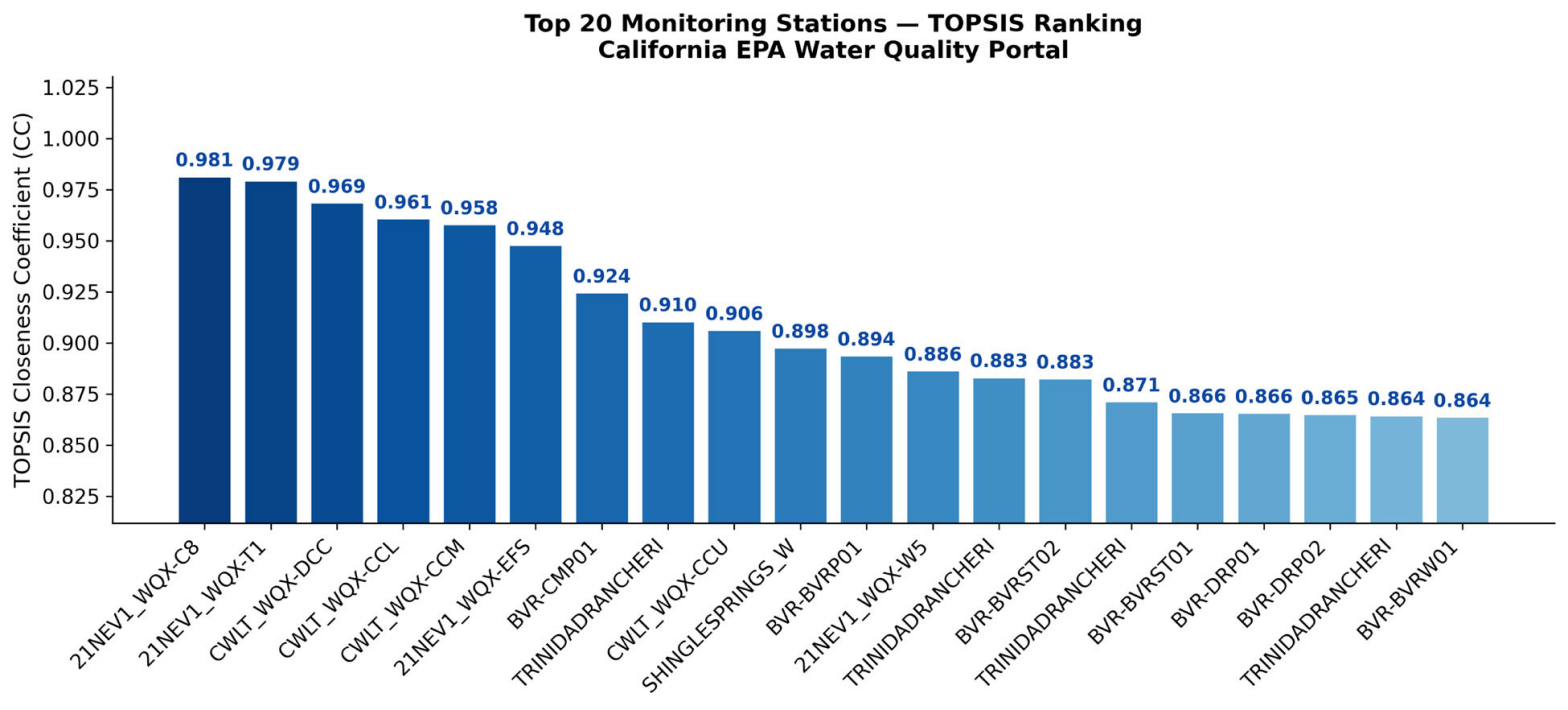
Top 20 water quality monitoring stations ranked by TOPSIS closeness coefficient. Nevada County (21NEV1) and Cache Creek Watershed (CWLT) stations dominate the top rankings, followed by Bear Valley Reserve (BVR) and Trinidad Rancheria EPA stations, reflecting the effectiveness of protected watershed management and conservation practices for maintaining high multi-parameter water quality performance

Figure 7 visualizes the top-ranked stations. The strong TOPSIS–VIKOR rank concordance (Table 3, VIKOR Rank column) confirms that station prioritization is robust across both methods: the top six positions are identical, and swaps in ranks 7–20 are modest in magnitude (e.g., CWLT_WQX-CCU: TOPSIS rank 9, VIKOR rank 7; BVR-BVRP01: TOPSIS rank 11, VIKOR rank 14; BVR-BVRST01: TOPSIS rank 16, VIKOR rank 26). The global Spearman concordance across all 347 stations ($\rho = 0.912$, *p* < 0.001) confirms the ranking is not an artefact of either method.

### Optimal resource allocation

Linear programming optimization identified an efficient resource allocation strategy concentrating resources on the top-performing stations, as shown in Table 4. The LP objective function value (0.9749) represents a 36.9% improvement over uniform distribution across all 347 stations (baseline $= \bar{C}_i = 0.712$, i.e., the mean TOPSIS closeness coefficient under equal allocation $a_i = 1/347$). Three stations received the maximum allowable allocation of 30% each, with the remaining 10% allocated to the fourth-ranked station.

**Table 4:** Optimal resource allocation for monitoring network (LP solution, $a_i^{\max } = 0.30$)

Rank	Station ID	TOPSIS CC	Resource allocation (%)
1	21NEV1_WQX-C8	0.9813	30.0
2	21NEV1_WQX-T1	0.9794	30.0
3	CWLT_WQX-DCC	0.9685	30.0
4	CWLT_WQX-CCL	0.9607	10.0
*All others*	—	—	0.0
Total	—	—	**100**.**0**

**Fig. 8:**
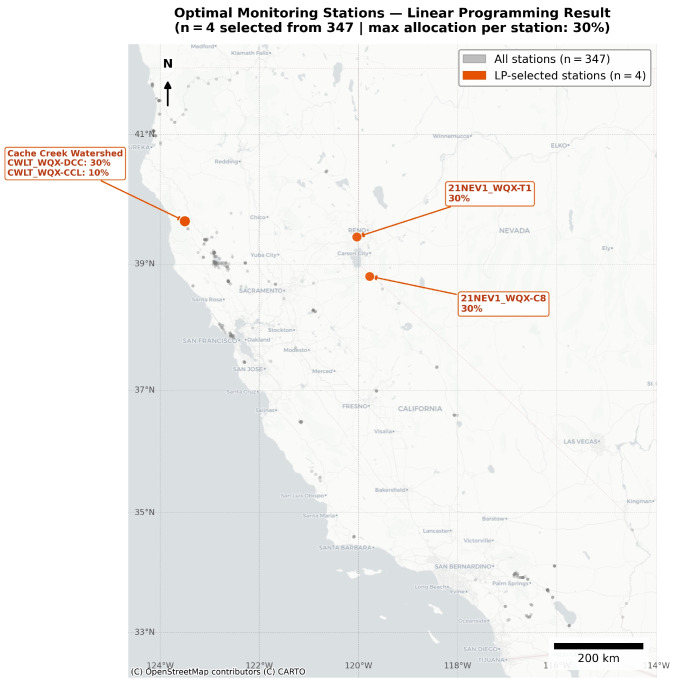
Spatial distribution of LP-optimal monitoring stations (orange markers) against the full California monitoring network (gray markers). Selected stations span diverse watershed types in northern California. Each label indicates station ID and allocated resource share. The spatial map enables assessment of geographic coverage and potential monitoring gaps under the optimized allocation

Figure 8 presents the spatial location of LP-selected stations across California, enabling assessment of geographic coverage and clustering. The selected stations are geographically dispersed across northern California, covering distinct watershed types. Sensitivity analysis varying $a_i^{\max }$ from 10 to 50% confirmed stable concentration patterns: resource concentration increases monotonically as the individual limit relaxes, with efficiency gains ranging from 32.5% ($a_i^{\max } = 10\%$, resources spread across 10 stations) to 37.7% ($a_i^{\max } = 50\%$, resources concentrated on 2 stations), all substantially exceeding the 36.9% achieved under the base case ($a_i^{\max } \!=\! 30\%$). The narrow range of improvement across constraint values (32.5–37.7%) confirms that the efficiency advantage of concentrated allocation is robust to policy choices about maximum individual station allocation, while reinforcing the need for practical minimum-coverage constraints.

## Discussion

### Methodological contributions and advances

This study demonstrates a fully reproducible, data-driven framework for water quality monitoring network optimization, addressing gaps in existing research identified in the literature review. The integration of CRITIC objective weighting, TOPSIS, and VIKOR ranking, and LP resource allocation provides a transparent decision support tool free from subjective bias (Li et al. 2024; Das and Das 2024).

Four key methodological advances characterize this framework: (1) *Corrected DO criterion*—treating DO as an optimal-range criterion (|DO−8 mg/L|) rather than a monotonic benefit eliminates a systematic error common in MCDA water quality studies (Dodds 2002), directly affecting CRITIC weights and TOPSIS rankings; (2) *Non-parametric correlation*—replacing Pearson with Spearman correlation in CRITIC computation appropriately accounts for the skewed, non-normal distributions confirmed statistically for all seven parameters; (3) *Physical plausibility filters*—removing 1106 impossible values (2.4%) prior to analysis ensures that statistical outputs reflect real environmental conditions rather than instrument artefacts; (4) *Dual MCDA validation*—TOPSIS–VIKOR rank concordance ($\rho = 0.912$, *p* < 0.001) confirms that station prioritization is robust across methodological choices, strengthening confidence in LP-based resource recommendations.

The weight distribution (range: 0.0932–0.1813) shows meaningful differentiation across criteria, reflecting that DO deviation and phosphorus carry substantially more independent information than turbidity under the Spearman-based CRITIC computation. This result highlights how criterion classification and correlation method substantially alter MCDA outcomes (Das and Das 2024).

### Interpretation of CRITIC weights and water quality implications

The CRITIC analysis reveals that DO deviation from 8 mg/L received the highest weight (0.1813), reflecting that many California stations deviate substantially from this ecologically optimal threshold—an information pattern obscured when DO is treated as a monotonic benefit criterion (Dodds 2002). Phosphorus (0.1804) ranked second, reflecting high station-to-station variability driven by heterogeneous agricultural and urban inputs across California watersheds.

TDS received a moderate weight (0.1308) reflecting its partial Spearman correlation with nitrate ($\rho = 0.46$), likely from shared agricultural sources (irrigation return flows simultaneously increasing salinity and nutrient concentrations), appropriately reducing its independent information content. This co-loading pattern is consistent with agricultural diffuse-source dynamics documented in surface water monitoring studies (Singh et al. 2024). Turbidity received the lowest weight (0.0932), suggesting that once DO, phosphorus, and pH are accounted for, turbidity provides less independent discriminatory power across this network. This finding is consistent with Das and Das (2024), who similarly reported low independent information content for turbidity relative to nutrient and oxygen parameters in a coastal groundwater CRITIC application. By contrast, Li et al. (2024) found turbidity to carry higher weight in the Liao River network, where erosion-driven suspended sediment constitutes the dominant pollution signal—illustrating how CRITIC weights are context-specific and directly reflect the dominant local stressors rather than universal parameter hierarchies.

### TOPSIS–VIKOR rankings and spatial patterns

Nevada County (21NEV1 network) dominates the top rankings, occupying four positions (ranks 1, 2, 6, and 12; CC = 0.9813–0.8864). This network monitors tributaries and reaches within the Sierra Nevada foothills of northern California, a region characterized by low agricultural intensity, limited urban development, and largely forested catchments under public and private conservation management. The consistent multi-parameter performance across four stations within the same network suggests that watershed-scale land-use controls, rather than station-specific factors, drive the observed water quality outcomes.

Cache Creek Watershed (CWLT network), managed by the Cache Creek Watershed Land Trust, occupies four consecutive positions in the top 10 (ranks 3, 4, 5, and 9; CC = 0.9685–0.9063). The clustering of four stations from a single coordinated monitoring program reflects systematic water quality management across the watershed, with active conservation easements and restoration work suppressing nutrient loading and turbidity. The contiguous ranking pattern further suggests spatial coherence in water quality conditions throughout the Cache Creek corridor.

Bear Valley Reserve (BVR), part of the University of California Natural Reserve System, contributes seven stations to the top 20 (ranks 7, 11, 14, 16, 17, 18, and 20; CC = 0.9245–0.8638)—the largest representation of any single network. As a protected research reserve with strict land-use controls and no agricultural or industrial activity, BVR stations consistently exhibit low contamination across all seven parameters. Trinidad Rancheria EPA (four stations, ranks 8, 13, 15, and 19) and Shingle Springs (rank 10) complete the top 20, reflecting the role of tribally managed environmental monitoring programs and regulated land management in maintaining above-average water quality.

The TOPSIS distribution (mean = 0.712, MAD = 0.063, CV = 12.6%) indicates moderate heterogeneity across the 347-station network, with clear differentiation between protected-area stations and agricultural or urban watershed stations. This pattern parallels findings by Li et al. (2024) in the Liao River Basin, where network evaluation using entropy-TOPSIS similarly revealed that stations in protected or minimally disturbed sub-basins consistently outperformed those in agricultural and industrial catchments, despite a different parameter set and weighting method. The dominance of tribal, reserve, and conservation-trust networks in the California top tier further aligns with global evidence that catchment-scale land use composition is a primary driver of multi-parameter water quality performance (Singh et al. 2024; Mendivil-García et al. 2024). The strong TOPSIS–VIKOR concordance ($\rho = 0.912$) confirms that this discrimination is methodologically robust and not an artefact of either ranking approach (Opricovic and Tzeng 2004).

### Resource allocation implications and policy considerations

The LP optimization recommended concentrating resources on four stations, achieving a 36.9% improvement in objective function value over uniform distribution ($\bar{C}_i = 0.712$). This efficiency gain reflects the genuine heterogeneity in water quality performance across the monitoring network (MAD = 0.063, CV = 12.6%): the LP exploits real inter-station quality differences to concentrate resources where they generate the greatest monitoring effectiveness. The spatial map (Fig. 8) confirms that LP-selected stations are geographically distributed across northern California rather than clustered at a single location, partially alleviating coverage concerns.

Practical implementation requires considering: (1) *Spatial coverage*—concentrated allocation may create monitoring gaps in distant watersheds where pollution sources exist but detection capability is reduced; (2) *Problem detection*—resources concentrated in high-quality areas may fail to detect emerging problems in lower-quality locations requiring intervention; (3) *Stakeholder equity*—communities near unmonitored stations may perceive inequitable environmental protection; (4) *Redundancy*—concentration creates single points of failure if equipment malfunctions or stations become inaccessible.

Modified optimization formulations could address these concerns by incorporating: minimum allocation requirements for different geographic regions, ensuring statewide coverage; explicit constraints on maximum distance between monitored locations; weighted objectives incorporating both quality maximization and problem detection probability; and multi-objective frameworks balancing efficiency, coverage, and equity explicitly.

### Comparison with alternative monitoring network design approaches

The integrated CRITIC-TOPSIS-LP framework offers several advantages over alternative monitoring network design approaches reported in recent literature. Compared to artificial intelligence-based optimization (Mendivil-García et al. 2024), the current approach provides greater transparency through explicit mathematical formulations, enabling stakeholder understanding and verification. While AI methods may identify complex nonlinear patterns, they often function as “black boxes” limiting interpretability essential for regulatory decision-making.

Relative to entropy-TOPSIS approaches (Li et al. 2024), CRITIC weighting better captures contrast intensity through standard deviation while entropy focuses primarily on information dispersion (Diakoulaki et al. 1995; Shannon 1948). For water quality parameters exhibiting skewed distributions (as confirmed for all seven parameters by Shapiro–Wilk tests), CRITIC’s Spearman-based computation provides more appropriate weight assignment. However, hybrid approaches combining multiple objective weighting methods demonstrate potential for enhanced robustness (Khan and Ayaz 2024; Gai and Guo 2023).

Zhou et al. (2023) demonstrated synergies from combining multiple statistical methods (correlation analysis, principal component analysis), WQI models, machine learning (random forest), and positive matrix factorization for comprehensive water quality evaluation. Their integrated approach enabled both overall condition assessment and pollution source apportionment, suggesting that combining CRITIC-TOPSIS with additional analytical techniques could further enhance monitoring network optimization. Specifically, integrating machine learning for parameter importance ranking (Wang et al. 2023) or positive matrix factorization for source tracking (Zhou et al. 2023) could complement the current framework.

Statistical risk assessment approaches provide alternative perspectives on water quality evaluation. Brito et al. (2024) developed a risk-based assessment methodology using six different risk measures (mean, variance, loss probability, entropy, mean excess loss, value at risk) with ranking aggregation methods, enabling identification of riskiest periods and locations. Integrating such probabilistic risk frameworks with CRITIC-TOPSIS ranking could enhance network design for early warning and rapid response applications.

Compared to pure optimization approaches without MCDA ranking (Thomas and Sela 2024), the integrated framework provides comprehensive station evaluation beyond simple mathematical programming. TOPSIS scores offer interpretable performance metrics facilitating stakeholder communication and validation, while pure optimization may yield technically optimal solutions lacking intuitive justification.

### Limitations and future research directions

This research exhibits several limitations, suggesting future research directions. First, despite physical plausibility filtering, residual data quality issues (calibration drift, sensor fouling, reporting inconsistencies) may affect analysis reliability. Future studies should implement automated quality control pipelines with real-time anomaly detection and cross-sensor validation to enhance data integrity upstream of MCDA analysis.

Second, temporal aggregation using station-level medians over 24 months obscures seasonal dynamics, diurnal variations, and episodic pollution events that are critical for early warning applications. Time-series analysis incorporating seasonal decomposition, event detection algorithms, and dynamic optimization formulations would provide richer temporal understanding supporting adaptive monitoring strategies (Poursaeid et al. 2025).

Third, the seven-parameter selection, while covering major regulatory indicators, omits emerging contaminants (pharmaceuticals, microplastics, per- and polyfluoroalkyl substances), habitat quality indicators, and biological monitoring endpoints. Expanded parameter sets incorporating these factors would enable more holistic water quality assessment aligned with contemporary environmental priorities (Singh et al. 2024).

Fourth, the optimal-range formulation for DO (deviation from 8 mg/L) represents a simplification; in reality, ecological thresholds vary by season, species assemblage, and altitude. Fuzzy membership functions accommodating parameter-specific and context-specific optimal ranges would better capture these nonlinear ecological relationships, and could be extended to pH as well.

Fifth, single-objective LP (maximize weighted TOPSIS scores) neglects explicit trade-offs with cost minimization, spatial coverage maximization, and problem detection probability. Multi-objective formulations using Pareto optimization or weighted goal programming would enable quantified trade-off analysis supporting informed decision-making under conflicting objectives (Zhu et al. 2018).

Sixth, the static optimization framework does not adapt as water quality conditions change, new pollution sources emerge, or monitoring technologies evolve. Dynamic optimization incorporating reconfiguration costs and forecasting models would support long-term network planning under uncertainty.

Finally, future work should evaluate alternative MCDA methods, including PROMETHEE and ELECTRE for robustness benchmarking, extending the methodological comparison initiated here with TOPSIS–VIKOR concordance analysis.

## Conclusion

This study presented an integrated MCDA framework for water quality monitoring network optimization using real EPA data from 347 California monitoring stations (January 2023–December 2024). Three key methodological decisions underpin the analysis: (1) DO was treated as an optimal-range criterion using |DO $- 8$ mg/L| rather than a monotonic benefit (Dodds 2002; 2) Spearman rank correlation was applied throughout CRITIC computation, justified by confirmed non-normal distributions (Shapiro–Wilk, *p* < 0.001) for all seven parameters; and (3) physical plausibility filters removed 1106 impossible values (2.4% of raw data) prior to any statistical analysis.

CRITIC weighting identified DO deviation (0.1813) and phosphorus (0.1804) as the most informative parameters under the corrected formulation, with turbidity receiving the lowest weight (0.0932) due to lower independent information content. TOPSIS analysis produced closeness coefficients ranging from 0.9813 to 0.3799 (mean = 0.712, MAD = 0.063), reflecting genuine discrimination across California’s diverse monitoring network.

VIKOR validation confirmed ranking robustness (Spearman $\rho = 0.912$, *p* < 0.001) across all 347 stations. Top-ranked stations consistently belong to protected or cooperatively managed watersheds, reflecting that land-use controls at the catchment scale—rather than station-specific factors—are the primary driver of multi-parameter water quality performance.

LP optimization achieved a 36.9% improvement over uniform allocation ($\bar{C}_i = 0.712$), with four stations receiving non-zero resources and spatial mapping confirming the geographic distribution of selected stations across northern California. Spatial visualizations of both the full monitoring network and LP-optimal stations provide essential geographic context for interpreting network design recommendations.

The integrated CRITIC–TOPSIS–VIKOR–LP framework, relying exclusively on public EPA data with full methodological transparency, offers a reproducible foundation for evidence-based monitoring resource allocation applicable to other regional networks facing similar constraints. Future work should address temporal dynamics through seasonal disaggregation, expand to multi-objective optimization explicitly balancing efficiency and spatial coverage, and incorporate fuzzy optimal-range formulations for additional parameters beyond DO.

## Data Availability

All data derive from the EPA Water Quality Portal (https://www.waterqualitydata.us/), a publicly accessible repository. Python scripts for data collection, analysis, and optimization, along with processed datasets and complete results, are available as supplementary material to ensure full reproducibility.
